# Single-cell sequencing analysis characterizes common and cell-lineage-specific mutations in a muscle-invasive bladder cancer

**DOI:** 10.1186/2047-217X-1-12

**Published:** 2012-08-14

**Authors:** Yingrui Li, Xun Xu, Luting Song, Yong Hou, Zesong Li, Shirley Tsang, Fuqiang Li, Kate McGee Im, Kui Wu, Hanjie Wu, Xiaofei Ye, Guibo Li, Linlin Wang, Bo Zhang, Jie Liang, Wei Xie, Renhua Wu, Hui Jiang, Xiao Liu, Chang Yu, Hancheng Zheng, Min Jian, Liping Nie, Lei Wan, Min Shi, Xiaojuan Sun, Aifa Tang, Guangwu Guo, Yaoting Gui, Zhiming Cai, Jingxiang Li, Wen Wang, Zuhong Lu, Xiuqing Zhang, Lars Bolund, Karsten Kristiansen, Jian Wang, Huanming Yang, Michael Dean, Jun Wang

**Affiliations:** 1 BGI-Shenzhen, Beishan Industrial Zone, Beishan Road, Yantian, Shenzhen, 518083, People’s Republic of China; 2 CAS-Max Planck Junior Research Group, State Key Laboratory of Genetic Resources and Evolution, Kunming Institute of Zoology, Chinese Academy of Sciences (CAS), 32# Jiao-chang Road, Kunming, Yunnan, 650223, People’s Republic of China; 3 Graduate University of the Chinese Academy of Sciences, 19A Yuquanlu, Beijing, 100049, People’s Republic of China; 4 College of Life Sciences, Wuhan University, Luojia Hill, Wuhan, 430072, People’s Republic of China; 5 School of Biological Science and Medical Engineering, Southeast University, Sipailou 2#, Nanjing, 210096, People’s Republic of China; 6 State Key Laboratory of Bioelectronics, Southeast University, Sipailou 2#, Nanjing, 210096, People’s Republic of China; 7 Shenzhen Key Laboratory of Genitourinary Tumor, Shenzhen Second People’s Hospital, First Affiliated Hospital of Shenzhen University, Shenzhen, 518035, People’s Republic of China; 8 Department of Urology, Shenzhen Second People’s Hospital, Shenzhen, 518035, People’s Republic of China; 9 The Institute of Urogenital Diseases, Shenzhen University, Shenzhen, 518060, People’s Republic of China; 10 BioMatrix, LLC, 3029 Windy Knoll Court, Rockville, MD, 20850, USA; 11 Cancer and Inflammation Program, National Cancer Institute at Frederick, Building 560, Frederick, MD, 21702, USA; 12 School of Bioscience and Biotechnology, Guangzhou Higher Education Mega Centre, South China University of Technology, Panyu District, Guangzhou, 510006, People’s Republic of China; 13 Guangdong and Shenzhen Key Laboratory of Male Reproductive Medicine and Genetics, Institute of Urology, Shenzhen PKU-HKUST Medical Center, Peking University Shenzhen Hospital, 1120 Lian Hua Road, Futian District, Shenzhen, 518036, People’s Republic of China; 14 Department of Urology, Longgang Central Hospital, Shenhui Road, Longgang Town, Shenzhen, 518116, People’s Republic of China; 15 Institute of Human Genetics, University of Aarhus, Aarhus, 8100, Denmark; 16 The Novo Nordisk Foundation Center for Basic Metabolic Research, University of Copenhagen, Ole Maaløes Vej 5, Copenhagen, DK, 2200, Denmark; 17 Department of Biology, University of Copenhagen, Ole Maaløes Vej 5, Copenhagen, DK, 2200, Denmark

**Keywords:** Single-cell exome sequencing, Bladder cancer, Tumor evolution, Population genetics

## Abstract

**Background:**

Cancers arise through an evolutionary process in which cell populations are subjected to selection; however, to date, the process of bladder cancer, which is one of the most common cancers in the world, remains unknown at a single-cell level.

**Results:**

We carried out single-cell exome sequencing of 66 individual tumor cells from a muscle-invasive bladder transitional cell carcinoma (TCC). Analyses of the somatic mutant allele frequency spectrum and clonal structure revealed that the tumor cells were derived from a single ancestral cell, but that subsequent evolution occurred, leading to two distinct tumor cell subpopulations. By analyzing recurrently mutant genes in an additional cohort of 99 TCC tumors, we identified genes that might play roles in the maintenance of the ancestral clone and in the muscle-invasive capability of subclones of this bladder cancer, respectively.

**Conclusions:**

This work provides a new approach of investigating the genetic details of bladder tumoral changes at the single-cell level and a new method for assessing bladder cancer evolution at a cell-population level.

## Background

Bladder cancer (BC) is among the top ten most common cancers in the world; and transitional cell carcinoma (TCC) is the most common form, presenting in 90% of diagnosed BC [1]. Previous studies have indicated that the development of TCC involves multiple steps [2-4], but the key mutations and how this process occurs remain largely unknown. Clinical and genetic studies have classified TCC patients into two main categories: non-muscle-invasive TCCs (NMI-TCCs), which occur in approximately 70% of the patients and often carry mutations in the *FGFR3* and *RAS* genes; and muscle-invasive TCCs (MI-TCCs), which occur in approximately 30% of the patients and often carry mutations in the *TP53* and *RB1* genes [5]. MI-TCC, however, is the form that is associated with a higher mortality rate [3], which makes this form of BC, though less common, of greater concern for developing the means to assess and ultimately devising viable treatments.

Current information has indicated that there is a shared genetic pattern in TCCs among patient populations [6], but it has not yet been possible to apply this information to understand tumor formation within a patient. Moreover, the heterogeneous nature of the tumor and its contamination by infiltrating ‘normal’ cells further complicate cancer studies, since the functionally important mutations may only reside in a portion of the cells within a tumor sample and would be undetectable in heterogeneous tumor tissues. Given the heterogeneous nature of tumors both among patients and within tumors, understanding tumors at a cell-specific level may be a direct way for developing targeted ‘personalized’ therapies for bladder cancer.

It is now feasible to gain greater insight into cellular selection within the tumors given the technical development of large-scale data acquisition and genome analysis, including the emergence of new methods of genome sequencing for copy-number genetic analyses [7] and single nucleotide analyses at the single-cell level [8,9]. However, there is currently no study that attempts to place the timing of key mutations within the development history of the tumor to infer their potential roles in tumorigenesis at the single-cell level, which is of great importance in developing effective cellular targeted therapies in personalized medicine.

Here we present results from single-cell exome sequencing (SCS) and analyses of a MI-TCC. The sequence data revealed the complexity of the genetic patterns within this tumor and identified the presence of genetically different tumor cell types within the tumor tissue. In addition, by placing the timing of key mutations within the development history of the tumor, we discovered candidate cancer-associated genes that might serve to drive not only the initiation of carcinogenesis, but also subsequent cell lineage development that may be involved in cancer progression.

## Data description

We obtained samples of fresh tumor (standard surgery of bladder cancer: >80% tumor cells) and para-carcinoma tissue from a 57-year-old male with MI-TCC of the bladder classified as stage II (T_2_-N_0_M_0_) ( Additional file 1: Figure S1, see Methods for details).

We carried out single-cell exome sequencing on individual cells from these samples as described in [8]. Briefly, we gently disrupted the tissue by collegenase I and IV, and randomly selected single cells from the tumor tissue and normal adjacent tissue. Exome capture was performed on the whole-genome amplification (WGA) products of each cell. The resulting libraries were then subjected to second-generation sequencing (see Methods for exome capture and sequencing details). To drastically reduce errors in the subsequent analyses, cells were discarded if they had <70% coverage of the exome targets or a significant false heterozygous rate across the X chromosome due to amplification and/or hybridization failures. With a total of 66 cells sequenced, 44 single cells from the tumor tissue (hereafter referred to as BC cells) and 11 from the normal adjacent tissue (hereafter referred to as BN cells) were qualified and selected for subsequent analyses ( Additional file 2: Table S1). The average sequencing depth in exome regions of the qualified single cells was 40-fold, compiling a comprehensive dataset of approximately 2,200-fold coverage from all cells, which enabled the genotype calling for the majority of sites in the exome regions [10]. We achieved an average of 88.6% whole-exome coverage of all qualified single cells ( Additional file 2: Table S1) and covered more than 60% of the target region greater than 5× sequencing depth in all cells ( Additional file 3: Figure S2C-D).

In addition to single-cell exome sequencing, we also sequenced the whole exome of bulk DNA from the same bladder cancer tissue with 137× coverage and the normal bladder tissue with 28× coverage to use as a control for evaluating the data quality of our SCS ( Additional file 3: Figure S2A-B Additional file 2: Table S1). Data described here is available in the NCBI Short Read Archive [SRA051489] and *GigaScience*[11].

## Analyses

### Variation calling and quality assessment

We carried out sequencing data evaluation, somatic mutation calling, and additional bioinformatics analysis as described by a pipeline described in [8,9], with minor modifications as detailed in Additional file 4: Figure S3.

Using these data, we first determined the percentage of alleles that were missing due to errors introduced by WGA and exome capture in each single BN cell, and found that approximately 40% of the heterozygotes had one allele dropout (ADO) ( Additional file 5: Table S2). This performance was comparable to previous work [8,9,12]. Additionally, we took the ADO rate into account for all subsequent analyses to ensure the accuracy of our findings.

We also examined the percentage of alleles in the homozygous samples that were false-discovery events due to errors or artifacts during SCS. The false discovery rate (FDR) was very low, at 6.7 × 10^-5^, which was comparable to that of conventional tissue sequencing using the same sequencing platform in a previous report [13]. We further assessed FDR by examining the sequence generated from the mitochondrial DNA. While not specifically targeted on the exome-capture array, sufficient mitochondrial DNA became captured to cover the 16,561 bp genome by 10-100× or more. The aligned sequence reads of mitochondrial DNA for each single normal cell were analyzed by sequence analysis/map (SAM) tools [14] to generate predicted variants. For 11 single normal cells, the predicted variants were manually inspected, and sites with fewer than five mutant reads were discarded. This resulted in seven mutations identified, with no two cells having the same mutation. Assuming all were false positive, this gave an FDR of 2.6 × 10^-5^. In total, the false discovery analyses provided very strong evidence that when multiple single cells had the same mutation, those variants were true somatic mutations.

We then ascertained somatic mutations in the tumor cells from SCS as noted in [8,9]. A somatic mutation was defined as a site that was consistently called homozygous in all BN cells (with a minimum of six read-coverage BN cells), but had mutant in less than three BC cells. We set this specific threshold for cell numbers to eliminate errors and artifacts from the SCS process: the required number of BN cell was based on a binomial test using our ADO ( Additional file 6: Figure S4BAdditional file 5: Table S2), qualified BN cell number, and whole-exome size; the required number of BC cells was determined based on a binomial test with the false discovery ( Additional file 6: Figure S4A), the qualified BC cell number, and the whole-exome size.

In total, 443 somatic mutations were identified from a single-cell exome and its 100 bp flanking regions (Table 1Additional file 5: Table S2), of which 146 were nonsynonymous mutations in a total of 205 somatic mutations in exons. We randomly selected 17 predicted genotypes (in 17 mutation genes), and with being able to amplify a total of 54 DNA fragments in randomly selected 33 cancer cells and three fragments in one normal cell (only limited DNA products for normal cells) as control, 54 (including all three in the normal cell) of the fragments (94.73%) and 100% of predicted genes were confirmed by PCR-Sanger capillary sequencing, supporting that our experiment and mutation calling pipeline used in this study was of high confidence. In addition, the vast majority of these somatic mutations (374 in 443, or 84.42%) ascertained from single cells were also supported by at least one mutant read from sequencing read data of bulk tissue DNA (BC tissue) ( Additional file 5: Table S2B), which further indicated that the mutation calls in single cells were mostly true positives after amplification errors were efficiently removed. Mutational spectrum in this tumor was dominated by C:G >T:A transitions, equivalent to a prior sequencing study of TCC [6]. Of note, none of these mutations appeared in the commonly mutant MI-TCC genes, *TP53* or *RB1*. Nonetheless, we observed significant enrichment of loss of heterozygosity and copy number variations [15] in Chromosome 9 and 11 ( Additional file 7: Figure S5), which were commonly mutant in MI-TCC [2,3], reconfirming this TCC was muscle-invasive.

**Table 1:** Summary of somatic mutations called in single-cell exome sequencing

**Mutant alleles observed in matched tissue sequencing**	**Total N (%)**	**CDS**			**3′UTR N (%)**	**5′UTR N (%)**	**Intron N (%)**	**Intergenic N (%)**
		**NS N (%)**	**S N (%)**	**NS/S**				
Yes	374(84.42)	133(91.10)	54(91.53)	2.46	13(92.86)	7(87.50)	161(77.40)	6(75.00)
No	69(15.58)	13(8.90)	5(8.47)	2.60	1(7.14)	1(12.50)	47(22.60)	2(25.00)
Total	443(100)	146(100)	59(100)	2.47	14(100)	8(100)	208(100)	8(100)

Note: ‘NS/S’ indicated the rate of the nonsynonymous mutation count to the synonymous one in CDS region.

CDS, Coding sequence; NS, Nonsynonymous; S, Synonymous; UTR, Untranslated region.

In addition to the evaluation of the specificity for mutation calling, we also took advantage of SCS to estimate the frequency of mutant alleles in the cell populations within the MI-TCC. The cell mutant allele frequency from multiple single-BC cells was highly correlative (R^2^ >0.89) with the mutant-read frequency in all read-covered mutant sites in BC tissue ( Additional file 6: Figure S4C). This result indicated that the frequency estimation from single cells was accurate and the genetic analyses of these single cells can, on behalf of this MI-TCC, enable us to apply population genetics analysis to this cell population.

Of additional note, when we used conventional exome sequencing of the DNA from the whole tissue samples and then compared the consensus sequences from the BC and BN tissues, though with very high sequencing depth (137-fold) for BC tissue, we could only identify 134 (30.25% of 443) high-confidence mutations found in our single-cell analyses, which indicated that single-cell analyses had a much higher sensitivity for identifying rare mutations present in tumor tissues.

### Inference of tumor development by population genetics methods

The comprehensive data set and accurate estimation of allele frequency, that we obtained for the tumor and normal cell populations, provided us an unprecedented opportunity to apply population genetics analysis methods to decipher the genetic pattern of this bladder cancer development.

**Figure 1:**
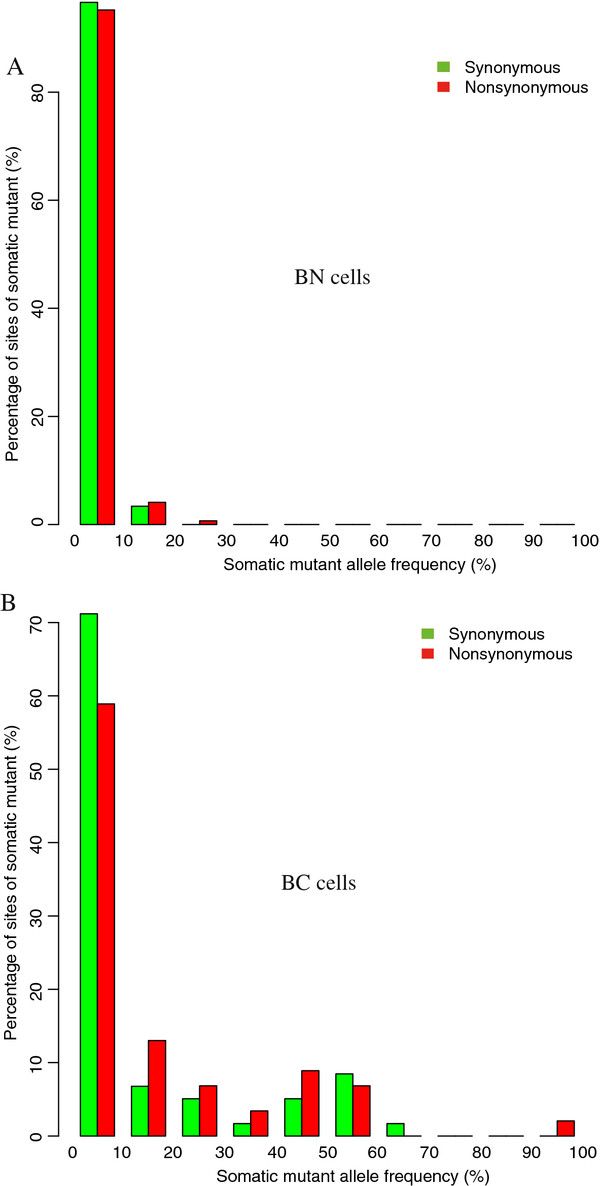
**Somatic mutant allele frequency spectrum (SMAFS) of synonymous (green) and nonsynonymous (missense and nonsense, red) mutants.** (**A**) Normal cell populations and (**B**) tumor cell populations. The allele frequency was binned to 10 columns; the first column denotes a frequency range from 0% to 10%, the second from 10% to 20%, and so forth.

To do so, we first derived the somatic mutant allele frequency spectrum (SMAFS) separately for the tumor and normal cells to gain a genetic landscape during the development process of this TCC (Figure 1). The SMAFS of normal cells showed that the somatic mutations identified in normal tissue did not spread across the population (nearly all mutations had a frequency of <0.1), suggesting that these changes were primarily cell-specific changes in terminally differentiated cells (Figure 1A). In contrast, the SMAFS of tumor cells showed an abundance of much higher frequency (> 0.1) mutant alleles in the cell population (Figure 1B). We observed a peak of the tumor SMAFS at around 50%-frequency (p-value = 9.0 × 10^-7^, Pearson’s goodness-of-fit test to a hyperbolic model), which was likely due to an excess of heterozygous mutations (where one allele was mutant and the other allele was wild type) in the majority of the tumor cells. This indicated that this TCC most likely originated from a single ancestral tumor cell (a cancer stem cell) that contained heterozygous mutations. Thus, all of the tumor cells that were descendants of this ancestral cell would contain the same common heterozygous mutations, thereby resulting in the observed 50%-frequency spectrum peak.

In addition, other somatic mutations outside the 50%-frequency peak displayed a hyperbolic decay in counts with mutant allele frequency increases, which is a characteristic of cell population expansion with somatic mutation accumulations under a neutral model— mathematically similar to a population genetics model proposed at the individual human level [16]. This suggested that the ancestral tumor cell acquired a growth advantage over the other cells and expanded from this event forward.

To assess whether the mutations under selection during tumorigenesis occured by tumor SMAFS, we found the nonsynonymous mutations had a frequency distribution of shift to the high frequency (right in the figure), in other words, they had a significant excess (p-value = 0.02, Fisher’s test) of higher frequency (> 0.1) mutations compared to the identified synonymous mutations (Figure 1B). This shift was also seen after excluding the 50%-frequency spectrum peak (40%-60%). Given that the synonymous mutations were under neutral selection and thus not conferring functional impacts, the above observation gave a signature that the somatic mutations were generally under positive selection in this MI-TCC. This suggested that the common ancestral tumor cell acquired characteristics of the tumor and had a continually proliferative advantage over the normal cells during the tumor development process. This constant selective pressure would provide a similar means for specific subclones of the tumor cells to gain additional mutations which provided a higher growth or survival advantage over other tumor cells. These beneficial growth conferring mutations have been defined as driver mutations [17], even though they may not be the triggering events, but instead were acquired during tumor progression.

We next subjected the identified mutations to a principal component analysis (PCA) to characterize the genetic heterogeneity of tumor and normal cells ( Additional file 8: Figure S6). Here, the first vector clearly differentiated the tumor cells from normal cells into two distinct clusters, which indicated there was no contamination of normal cells during tumor cell selection from the dissected tumor tissue, or vice versa. The eigenvector positions of all the normal cells and normal tissue showed they were nearly identical, also indicating no contamination in normal tissue samples. In contrast, the tumor cells showed considerable diversity across the all principal vectors (first to fourth vectors), which indicated this MI-TCC was heterogeneous and also supported our conclusions that the tumor cell population expanded during positive selection on these specific mutant cells.

### Identifying key mutant genes in TCC

**Figure 2:**
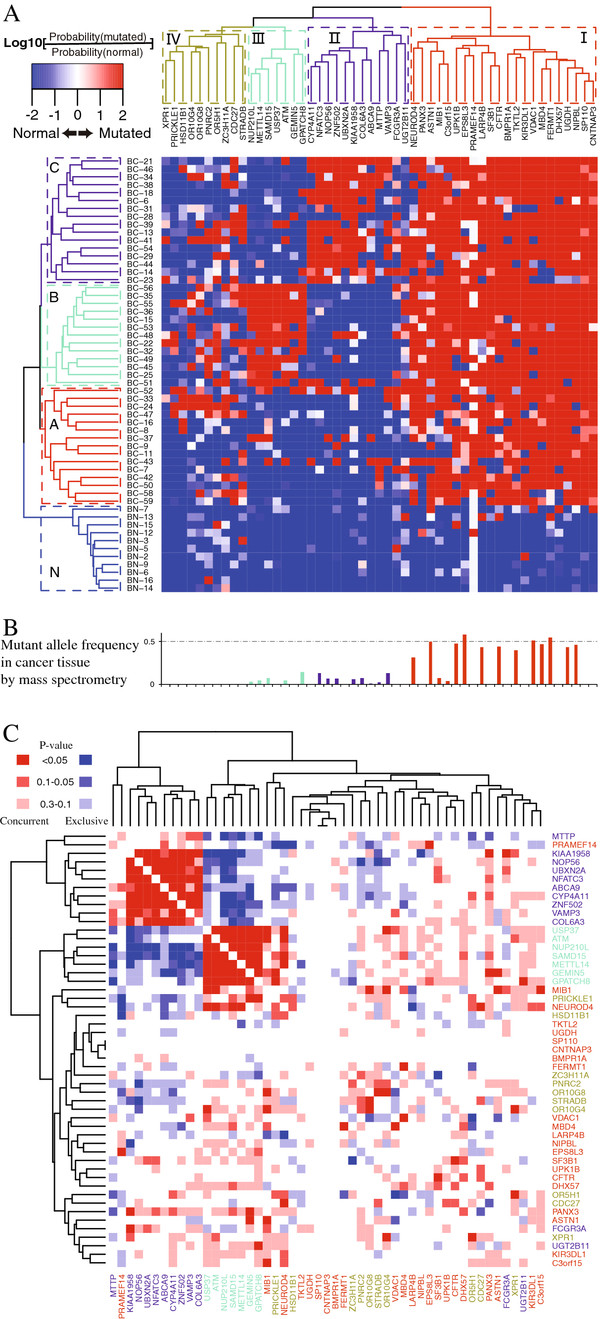
**Clonal structure of tumor and normal cells.** (**A**) Clonal structure of tumor and normal cells (rows) profiled in a heat map by nonsynonymous mutant genes (columns). Based on sequencing data and taking uncertainty in allele observation caused by allele dropout and binomial noise, the likelihood ratio for being not mutant was calculated for every gene in every cell. A profiling color of red meant a gene of likely mutant in the cell, while blue meant not mutant. Three major cell subclones were identified in tumor cells: 1) Clone A, with concordant mutant genes in Group I; 2) Clone B, with concordant mutant genes in Group I and Group III; 3) Clone C, with concordant mutant genes in Group I and Group II. Normal cells were clustered together (Clone N), free of mutations in all the three gene groups. (**B**) Somatic mutant allele frequency of certain genes in cancer tissue were detected by mass spectrometry. We detected 27 genes, in the three subclones incancer tissue by mass spectrometry (MassARRAY Analyzer) genotyping. (**C**) Concurrent and exclusive mutation analysis of mutant genes in tumoral cell population. Concurrent and exclusive mutation analysis was performed with two Perl packages [41,42]. The result was a concurrent and exclusive p-value between each two selected genes, indicated as depths of color. Note: a p-value ≥0.3 was indicated as white.

To identify key genetic changes within this TCC, we looked for genes that were commonly mutated, and then placed the timing of these changes within the development history of the tumor to infer their potential roles in tumorigenesis. With numerous mutations ( Additional file 5: Table S2), SCS allowed us an unprecedented opportunity to profile 146 nonsynonymous mutations (in 113 genes, see Additional file 5: Table S2B), to decipher the composition of tumor heterogeneity (Figure S6, Additional file 5: Table S2C), and to understand the clonal structure of tumor cells. Using heat map and its matched cell-lineage tree analyses (see details in Methods), we clustered the tumor cells into three identifiable subclones, each with distinguished signatures by different sets of genes (Figure 2A). The subclone-specific genes were also supported by concurrent and exclusive mutation analysis (Figure 2C), for the two set of subclone-specific genes were almost all (17/19) mutually exclusive with each other. To further validate the subpopulations by an alternative technology, we detected 27 genes, which were randomly selected in the three subclones, with cancer tissue by mass spectrometry (MassARRAY Analyzer) genotyping. The 92.59% (25/27) mutant allele frequency in cancer tissue fitting with cell frequency in heat map, in the view of most cells with heterozygous mutations, reconfirmed high confidence of our heat map clustering (Figure 2B).

**Figure 3:**
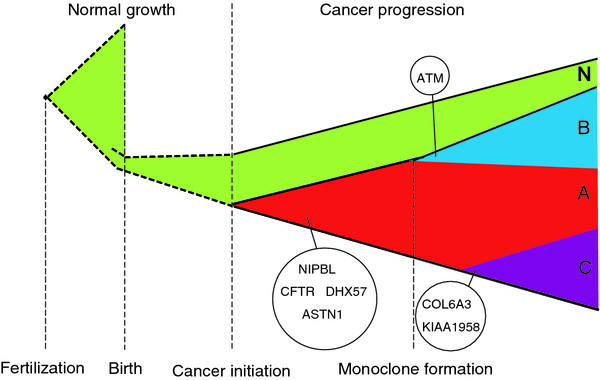
**Schematic diagram of cancer initiation and progression of this TCC patient.** The cancer initiation and progression was placed within the life history of this TCC patient. A total of eight recurrent genes, involved in three clones, are indicated in the schematic diagram. A (red), B (blue), C (purple) represent Clone A, B, C in the cancer cell population, respectively; N represents Clone N (green) of the normal cell population.

Almost all cells in the three subclones (Clones A, B, C, and their inferred schematic diagram of clonal formation in Figure 3 that would be discussed in next section) carried mutations in a set of 22 common genes (Group I). The presence of these commonly mutant genes, which should include the driver gene or genes that initiated tumorigenesis, in the cell populations further suggested these subclones were derived from a common ancestral tumor cell.

In addition, we also found two emerging subclones (Clones B and C) that separately obtained additional clone-specific mutant genes (7 and 12, respectively for Clones B and C in Figure 2A) that occurred subsequently to initiating events in tumor progression. Although numerous evaluations with varying mutation rates in tumor tissues and normal tissues [18-20], we conservatively assumed a mutation rate of 5 × 10^-9^ per base pair per cell generation, given that cancer tissues did not have a lower mutation rate than adjacent normal tissues. Thus, the divergence time between clones should be less than 40 generations. Hence, the two emerging subclones appeared to have originated late in the tumor history, and should only make up a small proportion of all the cells by random. However, Clones B and C each represent approximately 35% of the tumors’ cells, which was significantly larger than expected. This suggested the two subclones were undergoing positive selection over original Clone A, revealing the subclone-specific genes were not all passenger mutations, but instead included mutations that conferred additional growth or survival advantages. Thus, subclone-specific genes were likely to be equally important in understanding the complex makeup of this tumor.

Next, we surveyed the identified commonly mutant genes in an additional cohort of 99 TCC patients by the conventional exome sequencing [6] ( Additional file 9: Table S3), to determine whether these mutant genes were all unique to this patient or exist recurrently as mutant genes which would more likely to be candidate cancer genes. We found four genes, out of the 22 commonly mutant genes (Group I), also had non-silent mutations in at least three other TCC patients, including *CFTR* (mutant in seven patients), *NIPBL* (mutant in five patients), *ASTN1* (mutant in four patients) and *DHX57* (mutant in three patients), which were all novel findings of this study. These mutant genes in the derived ancestral tumor cell may have complementary rather than overlapping functions, and then cooperate to confer an overall growth or survival advantage. Of interest, the known functions of these recurrently mutant genes were diverse. For example, *NIPBL* was a cohesion complex regulator, playing a role in recruiting histone deacetylases to chromatin, and was the cause of most cases of Cornelia de Lange Syndrome that can lead to severe developmental anomalies [21], and was also identified in ovarian and other tumors [22]. Mutations in these chromosome-remodeling proteins may lead to epigenetic changes followed by deregulated gene expression and consequently, tumorigenesis [23]. In addition, *CFTR* was a chloride channel and the cause of cystic fibrosis [24]; *DHX57* was a putative ATP-dependent RNA helicase truncated in this TCC tumor [25]; and *ASTN1* was a neuronal adhesion molecule shown to be mutant in previous tumor studies [26,27].

We also identified three recurrently mutant genes, which were all also first found in this study, among the subclone-specific genes that may serve as potential driver genes unique to clones B and C. Among Clone B, *ATM* was mutant and found recurrently altered in five other TCC patients. *ATM* was a known tumor suppressor that played a key role as a cell-cycle checkpoint kinase in response to DNA damage [28,29]. From Clone C-specific mutant genes, *COL6A3* and *KIAA1958* each recurred in four additional patients. *COL6A3* encoded a collagen protein reported to have significant changes in expression level in certain tumor tissues [30] and was a putative pancreas cancer biomarker [31]. The role of *KIAA1958*, however, remained uncharacterized, but its potential role made it an interesting candidate for future analyses in tumors.

To further assess the likelihood of these seven recurrent genes being important to TCC development of this patient, we also scored the genes with Q-score by cancer driver prediction [8,32] that believed driver genes were likely to contain significantly more nonsynonymous mutations than background mutations. We observed that four genes (*CFTRASTN1DHX57* and *KIAA1958*) had a Q-score higher than or close to 1 (10% false discovery rate). Although not all Q-score genes were higher than 1 ( Additional file 10: Figure S7), we still believed all these recurrent genes were likely to be of great importance in TCC, given that the key importance of a driver gene depends on its functional impact more than just accumulation of more and more nonsynonymous mutations.

## Discussion

Here we carried out deep exome sequencing of individual cells from both tumor and adjacent normal tissues of an MI-TCC patient. Overall, the genetic profile of gene mutations in this MI-TCC indicated that the genesis of this tumor was multi-factorial, though it did not include the typical genetic signatures found in many other TCC tumors. The data from the single-cell exomes allowed us to successfully apply a population genetics analysis using the individual cells and provided evidence that this MI-TCC had a monoclonal origin. With comparison of SMAFS analysis between tumor and normal cells, we discovered the tumor cells suffered constant positive selection and accumulated driver gene mutations during both the initiation and progression processes. We had successfully deciphered the subclonal structures and cell-lineage trees of this complex tumor tissue, and identified several putative driver gene candidates using additional tumor samples from a 99-patient cohort. Anchoring these mutant genes to cell-lineage tree branches revealed mutually exclusive subclone-specific driver gene candidates, which may provide an insight into how a tumor evolves into the difficult to treat metastasis.

Given the importance for understanding tumor development mechanisms for designing workable cancer treatments, several models have been proposed to characterize this complex process. These include the clonal evolution model [33], the mutator phenotype model [34], and the stochastic progression model [35]. With the data from our single-cell exomes, we reconstructed the developmental history of this tumor and its subclones (Figure 3), providing new information relevant to these models. Our data indicated this tumor had a monoclonal origin from an ancestral cell with multiple mutant driver gene candidates that could generate the hallmarks of cancer [36], which fit best with the clonal evolution model. The only ambiguity in our defined tumor development process, however, was whether the inferred common ancestral cell represented the initial tumor cell, or whether it emerged after an earlier tumor initiation event and developed growth/survival advantages over other early tumor cells via clonal selection. Further studies on early-stage tumor tissues would serve to distinguish between the two scenarios. Of additional interest, we found certain mutations that function in genome stability and DNA repair in this tumor, suggesting this MI-TCC would also be compatible with the mutator phenotype model, as several driver genes underlying tumor initiation followed by clone-specific expansion. The fact that our data fit both the cancer evolution and mutator phenotype model suggests that a single model may not serve as a suitable model for all types, or even one type, of cancer.

In this study we carried out genetic analyses of individual cells collected from a whole tumor, rather than from specific tumor coordinates. Thus a clear next step, given distinct genetic differences within the tumor [37], is to obtain a set of single cells from different tumor quadrants and carry out similar cell population analyses – also beneficial for designing more effective drugs and efficient treatments. Defining the genetic changes and the evolution of such changes within these different components of a tumor may indicate the presence of different types of selective pressures, or of specific genetic mutations that are important to areas of tumors that are interacting with separate types of cellular microenvironments.

## Methods

### Case report and sample collection

The patient was a 57-year-old male, diagnosed with primary muscle-invasive transitional cell carcinoma (MI-TCC) of the bladder according to the 2004 World Health Organization (WHO)/ International Society of Urological Pathology (ISUP) grading systems. The details of diagnosis were as follows ( Additional file 1: Figures S1 and Additional file 3: Figure S2): a 7 × 4 × 4 cm papillary tumor in the trigone of the urinary bladder, invaded the bladder wall – the cut surface was grey-white and brittle, and classified as stage II (T_2_-N_0_M_0_) under microscopic examination. Tumor invasion into shallow muscularis of bladder wall was observed, but it did not penetrate deep into the muscularis and serous layer. Furthermore the tumor did not invade the prostate tissue or cause a bilateral ureteral obstruction, and there was no metastasis in six pelvic lymph nodes. The hematoxylin eosin-stained sections prepared using the cancerous tissues were microscopically evaluated by two independent pathologists ( Additional file 3: Figure S2). Afterward the primary tumor sample and matched para-carcinoma tissue were obtained from a surgery-resected sample from this MI-TCC patient at the Peking University Shenzhen Hospital. Informed written consent was obtained from the study participant. The studies were conducted in accordance with the Declaration of Helsinki II and were approved by the local Ethical Committees.

### Single-cell isolation and WGA

Every step during the experiment was reduced to a strict minimum. With sufficient dispersion and cascade dilution of cells, single cells were randomly isolated from collagenase I and IV digested tumor or para-carcinoma tissues into PCR-ready tubes using an inverted microscope and a self-made mouth-controlled, fine hand-drawn micro-capillary pipetting system. The single cell isolation was visually confirmed by photograph under microscope. Afterward, WGA of single-cell DNA were performed using the REPLI-g® Mini Kit (Qiagen GmbH, Hilden, Germany) according to the manufacturer’s instruction, using a no cell reaction as a negative control, and a reaction of human tissue genomic DNA as positive control.

### Quantitation and genome-integrity assessment of the WGA products

The DNA concentration of the WGA products were measured with highly sensitive fluorescence-based Quant-iT^TM^ assays using the Qubit^TM^ Quantitation Platform (Life Technologies, Invitrogen, Carlsbad, CA, USA) according to the manufacturer’s instruction. The genomic integrity of the qualified products (> 60 ng/μl) were then assayed with their amplification represented by 10 housekeeping-gene-PCR tests, and the 10 genes (*PRDX6*, *RPL37a*, *ADD1*, *ARHGEF7*, *EIF2B2*, *PSMD7*, *PSMB6*, *MC2R*, *BCAT2*, and *ATP5O*) were interspersed across 10 different chromosomes. Afterward, WGA products of best performance from both housekeeping PCR (≥8/10) and Qubit assays (> 60 ng/μl) were selected for downstream experiments. Every step was performed along with a sample of genomic DNA from human tissue as a positive control and a no template reaction as a negative control, respectively.

### Whole exome capture and sequencing of samples

High molecular weight genomic DNA was extracted from the primary tumor and matched para-carcinoma tissue, respectively. And for both DNA samples (single cells and matched tissues), whole exome capture was accomplished based on liquid phase hybridization of sonicated 2 μg genomic DNA to the bait cRNA library synthesized on magnetic beads using the SureSelect® Human All Exon 50 Mb kit (Agilent Technologies, Santa Clara, CA, USA) according to the manufacturer’s protocol. The captured targets were subjected to massive sequencing using the Illumina HiSeq2000 with the paired-end 100 bp read option, according to the manufacturer’s instruction.

### Public dataset access

Human (*Homo sapiens*) reference genome sequence (Hg18) and its annotation files (e.g., dbSNP v128) were downloaded from the University of California Santa Cruz Genome Bioinformatics site [38]. The targeted region files of exome capture were downloaded from the Agilent Technologies website [39].

### Reference-guide genome assembly

SOAPaligner/SOAP2 version 2.20 was used to align all short sequencing reads to the Hg18 reference genome with a maximum of two mismatches and non gap parameters. The insert size distribution of each library was checked by Eland contained in the Illumina Pipeline, and thus the insert size range was set for SOAPaligner.

### SNP calling

All reads uniquely mapped to exome region and 100 bp-flanking regions were selected for SNP calling. SOAPsnp version 1.03 was used for calculating the likelihood of each cell genotype and mixed tissue. In each sample, putative SNPs were filtered based on the following criteria: (1) a Q20 quality cutoff; (2) at least five reads; (3) a p-value >0.01 (that means no significant difference between the sequencing quality of the two alleles in a heterozygous genotype); (4) a 5 bp distance from each other; and (5) 1/3 ~ 3 variation between quality score of two bases in heterozygous sites. And the final SNP data of the filtered SNP in each cell was combined.

### Evaluation of ADO rate

With the final SNPs, the ADO ratio is defined as the random non-amplification of one of the alleles present in a heterozygous sample.

We first defined a background dbSNP subset, which was read-covered in both tissue sequencing of the tumor and matched normal among dbSNP. We then determined the heterozygous coverage ratio per sequencing depth, which was calculated by dividing the heterozygous number of this sample by the total read-covered number in this background dbSNP subset, for single normal cell and normal tissue sequencing, respectively. We then calculated the relative FNR per depth in a single normal cell,

(1) $$Relative\;FNR\left(n\right)=\frac{\frac{HR\left(T,n\right)}{CR\left(T,n\right)}-\frac{HR\left(S,n\right)}{CR\left(S,n\right)}}{\frac{HR\left(T,n\right)}{CR\left(T,n\right)}}$$

*n* the sequencing depth

*T* the tissue sequencing

*S* the single cell sequencing

*HR* the heterozygous rate of one kind of sequencing under *n ×* sequencing depth

*CR* the read-covered rate of one kind of sequencing under *n ×* sequencing depth

We thus calculated the ADO of a single normal cell, which was the median of all relative FNR per depth on non-outlier depths (the outlier depth was the depth when the FNR in tissue sequencing at this depth was higher than the SCS). And finally we calculated the ADO in all whole-exome SCS as the mean of ADO of all normal cells.

### Evaluation of FDR

The FDR is defined as a false heterozygous site in a homozygous sample, which may be due to amplification, hybridization or sequencing errors. We chose a homozygous subset which was high-confidence (Quality-score = 99 and 95% confidence interval of distributive depth under Poisson distribution), and then we calculated the number of discrepant sites of single qualified normal cell in the homozygous subset. The FDR per cell was indicated as the number of discrepant sites divided by total covered number of high-confidence homozygous subset. And finally the FDR in all whole-exome SCS was indicated as the mean of all qualified normal cells.

### Identification and experimental validation of somatic mutations

To eliminate the random errors or artifacts induced by SCS, we built two binomial tests to detect high-confidence point somatic mutations (SMs) in capture regions. The putative SMs were filtered based on the following criteria: (1) homozygous normal in all normal cells (at least read-covered in six normal cells); (2) the homozygous genotypes in normal cells were consistent in normal tissue; (3) at least mutant in three cancer cells among a total of 44 qualified cancer cells.

The criterion (1) was set by a binomial test with ADO, qualified normal cell number, and whole-exome size to eliminate the random errors.

(2) $$\begin{array}{l} P(i)=C_{n}^{i}{p_{a}}^{i}{\left(1-p_{a}\right)}^{\left(n-i\right)}\\ P(i)\cdot S<1 \end{array}$$

*i* the normal cell number of read-covered for a specific mutation

*n* the total number of qualified normal cells

*p*_a_ the ADO

*P*(*i*) the probability under binomial distribution

*S* the whole-exome size

Then the criterion (1) was set according to the largest cell number *i* fulfills above inequation.

And the criterion (3) was set by a binomial test with FDR, qualified cancer cell number, whole-exome size to eliminate the random errors.

(3) $$\begin{array}{l} P(i)=C_{n}^{i}{p_{f}}^{i}{\left(1-p_{f}\right)}^{\left(n-i\right)}\\ P(i)\cdot S<1 \end{array}$$

*i* the cancer cell number of mutant of a specific mutation

*n* the total number of qualified cancer cell

*p*_*f*_ the FDR

*P*(*i*) the probability under binomial distribution

*S* the whole-exome size

Then the criterion (3) was set according to the largest cell number fulfills above inequation.

High confident SMs in BC cells were randomly selected for experimental validation. The loci of corresponding single cells were amplified, sequenced and analyzed by MassARRAY® Analyzer (SEQUENOM, San Diego, CA, USA).

### Correlation of SMAF between SCS and whole tissue sequencing

Correlation coefficient of determination is a goodness-of-fit measure for models based on the proportion of explained variance. The somatic mutant allele frequency in SCS was indicated as unfolded site frequency of mutant alleles, and the somatic mutant allele frequency in tissue sequencing was indicated as read frequency of mutant alleles.

### SMAF

Cell population genetic inferences based on called (inferred) SNPs can lead to serious biases and possibly false inferences for the high ADO ratio for each cell. We have therefore developed a series of statistical techniques that can take uncertainty in genotype calls and allele frequency estimation into account. Instead of utilizing a Bayesian estimation-based method called site-frequency spectrum (SFS) on population individuals [10], we used this SFS to calculate the somatic allele frequency in each SM site of all BC and BN cells respectively, based on the same methods of estimating allele frequencies from reads in one site and additional estimating sample allele frequencies.

### PCA

To identify the most variable factors in classifying subgroups among single cancer cells, we utilized an R package, pcaMethods v1.12.0 [40], in performing the PCA based on the genotyping result at all somatic mutation sites on each single cell. Missing values were automatically estimated by probabilistic method within the R package.

### Heat map 2-dimension clustering

The 2-dimension heat map clustering of mutant genes and cells is based on the somatic mutations using R language. Color indicates the presence of tendency of mutant or normal, which was showed as log10 value of the relative probability of mutant to the probability of normal (non-mutant) after Bayes calibration by FDR, ADO and *a priori* probability from normal tissue.

For each mutant point site which is covered by sequencing reads in each cell, we first derived the mutant posterior probability by Bayes calibration with FDR, ADO and *a priori* probability from normal tissue. The values in heat map array and corresponding posterior mutant probability were calculated by the following formulas:

(4) $$P_{p}\left(A_{i}|O_{i}\right)=\frac{P_{r}\left(A_{i}\right)\times P\left(O_{i}|A_{i}\right)}{\sum_{i}P_{r}\left(A_{i}\right)\times P\left(O_{i}|A_{i}\right)}$$

(5) $$H=\log_{10}\left\{\frac{P_{p}\left(NC|O_{i}\right)+P_{p}\left(CC|O_{i}\right)}{P_{p}\left(NN|O_{i}\right)}\right\}$$

*A*_*i*_ is one kind of the three genotypes (*NN*, *NC*, *CC* for homozygous normal, heterozygous mutant, homozygous mutant, respectively) in the site of each cell.

*O*_*i*_ is one observed genotype (*NN* or *NC* or *CC*).

$P_{p}\left(A_{i}|O_{i}\right)$ is the mutant posterior probability of *A*_*i*_ on the observation of *O*_*i*_.

*Pr*_(*Ai*)_ is the priori probability of *A*_*i*_ calculated from the corresponding site at normal tissue.

$P\left(O_{i}|A_{i}\right)$ is the probability of *A*_*i*_ calibrated by ADO and FDR.

*H* is the logarithmic value of the relative probability of mutant to the probability of normal used for heat map 2-dimension clustering.

The priori probability of *A*_*i*_ calculated as following formulas:

(6) $$P_{r}\left(A_{i}\right)=\{\begin{array}{l} P_{r}\left(NN\right)=\{\begin{array}{c} P_{n}\\ \frac{1}{2}\left(1-P_{n}\right), \end{array}\;\begin{array}{c} NN\;in\;normal\;tissue\\ NC\;in\;normal\;tissue \end{array}\\ P_{r}\left(NC\right)=\{\begin{array}{c} P_{n}\\ \frac{1}{2}\left(1-P_{n}\right) \end{array}\;\begin{array}{c} NC\;in\;normal\;tissue\\ NN\;in\;normal\;tissue \end{array}\\ P_{r}\left(CC\right)=\frac{1}{2}\left(1-P_{n}\right) \end{array}$$

And

(7) $$P_{n}=\{\begin{array}{l} \frac{1}{3},Quality\;Score<10\;or\;rank\;sum\;test\;p-value<0.05\\ 0.9,10\leq Quality\;Score<20\\ 0.99,20\leq Quality\;Score \end{array}$$

Note: Quality score and rank sum test *p-value* are the values from the files after sequencing read alignment by SOAP.

The probability of *A*_*i*_ was calibrated by ADO and FDR as following formulas:

(8) $$P\left(O_{i}|\mathrm{NN}\right)=\left(1-f_{\mathrm{hom}o}\right)\times C_{n}^{m}\times f_{p}^{m}\times {\left(1-f_{p}\right)}^{\left(n-m\right)}$$

(9) $$P\left(O_{i}|\mathrm{NC}\right)\{\begin{array}{l} P\left(NN|NC\right)=\frac{1}{2}\times f_{\mathrm{heter}}\times C_{n}^{m}\times f_{p}^{m}\times {\left(1-f_{p}\right)}^{\left(n-m\right)}\\ P\left(NC|NC\right)=\left(1-f_{\mathrm{heter}}\right)\times 1\\ P\left(CC|NC\right)=\frac{1}{2}\times f_{\mathrm{heter}}\times C_{n}^{\left(n-m\right)}\times f_{n}^{\left(n-m\right)}\times {\left(1-f_{p}\right)}^{m} \end{array}$$

(10) $$P\left(O_{i}|\mathrm{CC}\right)=\left(1-f_{\mathrm{hom}o}\right)\times C_{n}^{\left(n-m\right)}\times f_{p}^{\left(n-m\right)}\times {\left(1-f_{p}\right)}^{m}$$

*f*_hom*o*_ is the ADO in homozygous genotype.

*f*_*heter*_ is the ADO in heterozygous genotype.

*f*_*p*_ is the FDR in single cell sequencing.

If a site is not covered by reads in one cell, then we estimate its log10 value by averaging all the log10 values in other cells at this site.

The columns and the rows were also ordered as in the tree with sub clusters. And certain suspicious genes illustrated as red (likely mutant) in BN cells, which were defined as ≥1 BN cell containing value ≥0.5 (light red), in our raw data were filtered in heat map clustering. Having been tackled with FDR, ADO and other sources of error, we suggested that the remaining suspicious genes may due to paralogous alignment or false negatives under our model. Under extreme low FDR value, the false negatives here might derive from super high sensitivity of our heat map model to even a low number of error sequencing reads in a genotype, that is, if there exist two mutant reads in a total of 30 sequencing reads in BN cells, its adjusted heat map-array value was likely to higher than 1 (red) in our model. However, two error sequencing reads out of 30 reads is understandable in a current second-generation sequencing platform. Finally, the remaining 51 genes (yellow-colored genes in Additional file 5: Table S2B) were used for the heat map and its matched cell-lineage tree analyses.

### Concurrent and exclusive mutation analysis

Concurrent and exclusive mutation analysis was performed with two Perl packages [41,42]. The depths of color in array were the concurrent and exclusive p-value between each two selected genes.

### Function analysis of key genes

To analyze the functional impact of the somatic mutations in key genes, we first investigated the protein sequence of each key gene from the UniProtKB/Swiss-Prot database [43]. The amino acid changes were determined by the human genome annotation files from the University of California Santa Cruz Genome Bioinformatics site [38]. We then mapped the abnormal amino acid changes to the protein sequence of each gene and determined which domain had been altered. Further, altered signaling pathways were determined by mapping the key genes to the Kyoto Encyclopedia of Genes and Genomes (KEGG) database [44].

## Availability of supporting data

The raw sequence data in the fastq format from this study were deposited in the NCBI Short Read Archive under the accession number SRA051489 and alignments and genotyping data is available from *GigaScience*[11].

## Abbreviations

ADO, Allele dropout; BC, Bladder cancer; BC cells, Single cells from tumor tissue; BN cells, Single cells from the normal adjacent tissue; bp, Base pair; FDR, False discovery rate; MI-TCCs, Muscle-invasive TCCs; NMI-TCCs, Non-muscle-invasive TCCs; PCA, Principal component analysis; SCS, Single-cell sequencing; WGA, Whole-genome amplification; SMAFS, Somatic mutant allele frequency spectrum; TCC, Bladder transitional cell carcinoma.

## Competing interests

The authors declare that they have no competing interests.

## Authors’ contributions

JuW, MD and HY designed the study; LS, ZeL, KW, HW, XY, GL and LW performed the experiments; LY, XX, LS, YH, ST, FL, KMI, BZ and WX analyzed the data; JieL, RW, HJ, XL, CY, HZ, MJ, LN, LW, MS, XS, AT, GG, YG, ZC, JinL, WW, ZuL, XZ, LB, KK and JiW contributed the reagents, materials, and analysis tools; YL, XX, LS, YH, HY, MD and JuW wrote the manuscript. All authors read and approved the final manuscript.

## Supplementary Material

Additional file 1 **Figure S1.** Clinical information of this MI-TCC sample. (A). Photography gross organ of the MI-TCC sample after surgery. (B). Histology of the MI-TCC sample. A hematoxylin eosin-stained tumor aspirate smear of the TCC patient was showed. (PDF 660 kb) Click here for file

Additional file 2 **Table S1.** Sequencing data summary and allele dropout estimation in single-cell sequencing of TCC. (A). Qualified samples. Cell IDs with “BC” (Bladder Cancer) as prefix were cells from tumor tissue, and those with “BN” (Bladder Normal) from tumor-adjacent normal tissue. The “SC Mean” row was summary of all single cells while values were indicated with 95% confidence interval by “Mean ± SEM”. The last two rows were summary of data production from sequencing tumor tissue DNA (“BC-Tissue”) and tumor-adjacent tissue DNA (“BN-Tissue”). Mean depth was calculated by total aligned data mapped to exome capture target regions divided by the length of target regions. Coverage was calculated by length of target regions covered by at least one uniquely mapped read divided by the total length of target regions. (B). Filtered samples. The four cells covering >70% were filtered for significant high false SCS error, which was indicated as high false heterozygous rate across X Chromosome in the male patient. The rest of cells were filtered for low exome coverage (<70%). **(C).** Allele dropout (ADO) estimated by single normal cells. The mean was indicated by with 95% confidence interval by “Mean ± SEM”. Click here for file

Additional file 3 **Figure S2.** Fold coverage of target regions for single cells and matched tissues sequenced in the Discovery Screen. (A). The box plot depicted the distribution of mean coverage of tissue from cancer and matched normal tissue sequenced in the discovery stage. CT, cancer tissue; NT, normal tissue. (B). The box plot depicted the distribution of fraction of targeted bases covered by at least 1×, 5× and 10 × across the cancer tissue. (C). The box plot depicted the distribution of mean coverage of all cells from cancer and matched normal sequenced in the discovery stage. Lines in the two central boxes showed the medians, and lines outside the two central boxes showed the first and the third quartiles of the mean depths. SCC, single cells from cancer; SCN, single cells from normal. (D). The box plot depicted the distribution of fraction of targeted bases covered by at least 1×, 5× and 10 × across the 44 qualified cancer cells. Lines in the inner three boxes showed the medians, and lines outside the three boxes showed the first and the third quartiles. Click here for file

Additional file 4 **Figure S3.** Bioinformatics pipeline of single cell analyses. Click here for file

Additional file 5 **Table S2.** Somatic mutations in this TCC patient. (A). Summary of somatic mutation count in this TCC patient. (B). Annotation of somatic mutations in this TCC patient. (C). Somatic mutations in the single cell population this TCC patient. Click here for file

Additional file 6 **Figure S4.** Quality assessment of single-cell sequencing. (A) and (B). Relationship between estimated false discovery rate (FDR) / allele drop-out (ADO) rates with quality score and read depth after sequencing read alignment in SCS. The selected thresholds (Q20 with depth≥6) in a single cell would result in 0.41 ADO rate and 6.7E-05 FDR. (A). The FDR varied along with the sequencing depth and quality score respectively. (B). The ADO rate varied along with the sequencing depth and quality score respectively. (C). Correlation of frequency between single-cell mutant allele count and mutant allele count in whole tissue sequencing. The single-cell allele count was calculated from the haploids that harbor mutant alleles divided by the total number of haploids (number of cells times 2). The allele count in whole tissue sequencing was calculated by reads harboring mutant alleles divided by total reads covering a site. Click here for file

Additional file 7 **Figure S5.** Copy number variation (CNV) and loss of heterozygosity (LOH) analysis in tissue sequencing of TCC and its matched control across the targeted regions. The CNV and LOH were analyzed with ExomeCNV [15] with default parameters. The most outer ring showed the chromosome ideograms in a pter–qter orientation, clockwise with the centromeres in red. From inside to outside, each data track represented (without Chromosome X and Y): The middle cycle: log ratio of tumor and normal depth-of-coverage, with the segment mean in pink line, the region of gain highlighted in red, and the region of loss highlighted in blue; The inner cycle: the B-allele frequencies (BAF) from ExomeCNV output from tissue exome sequencing with the region of LOH highlighted in blue. Click here for file

Additional file 8 **Figure S6.** Principal component analysis (PCA) based on mutant alleles divided tumor cells and normal cells. Since normal cells were genetically extremely close to each other, they were not distinguishable and shown as one point. The exome of BC tissue (purple asterisk) and BN tissue (red tip) were also shown in the PCA to indicate averaged signals. Click here for file

Additional file 9 **Table S3.** A list of somatic mutations identified in the cohort of 99 TCC patients. Click here for file

Additional file 10 **Figure S7.** Driver gene prediction of the recurrent Genes of the TCC. The driver gene prediction analysis of the 7 recurrent genes was indicated as Q-score. The vertical axis was the Q-score, and the circle area indicated the cell mutation frequency. Click here for file
