# Supplementary figures and images for: Single-cell sequencing analysis characterizes common and cell-lineage-specific mutations in a muscle-invasive bladder cancer
Source: Gigascience. 2012 Aug 14;1:12. doi: 10.1186/2047-217X-1-12 (PMC3626503; doi:10.1186/2047-217X-1-12)

A

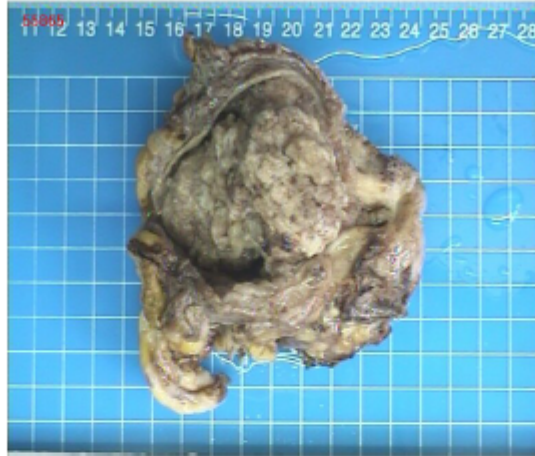

B

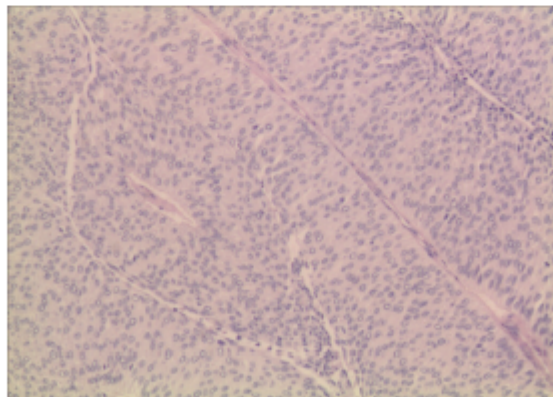

Supplement: Additional file 1 — Figure S1. Clinical information of this MI-TCC sample. (A). Photography gross organ of the MI-TCC sample after surgery. (B). Histology of the MI-TCC sample. A hematoxylin eosin-stained tumor aspirate smear of the TCC patient was showed. (PDF 660 kb) [file 2047-217X-1-12-S1.pdf]

A.

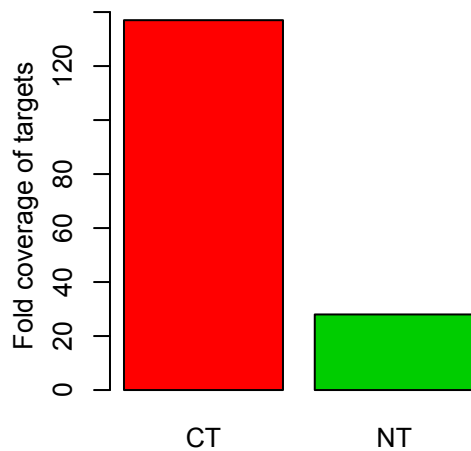

B.

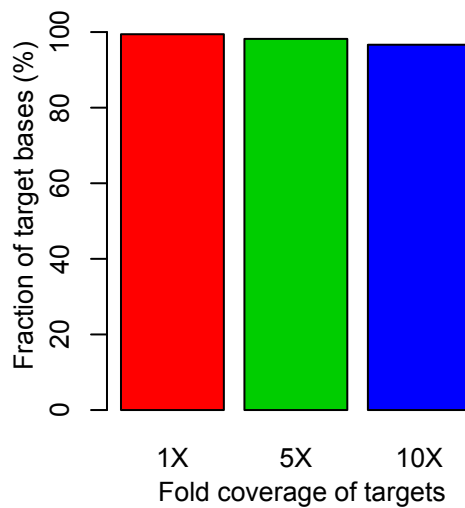

C.

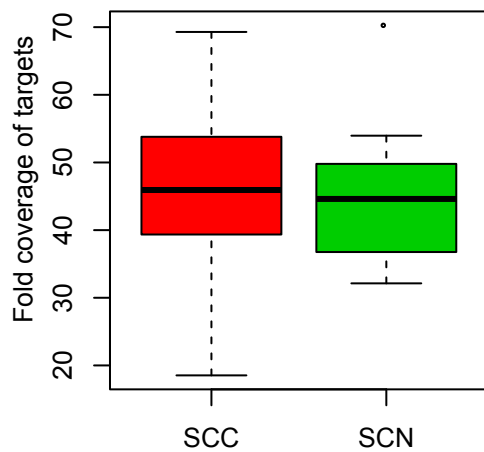

D.

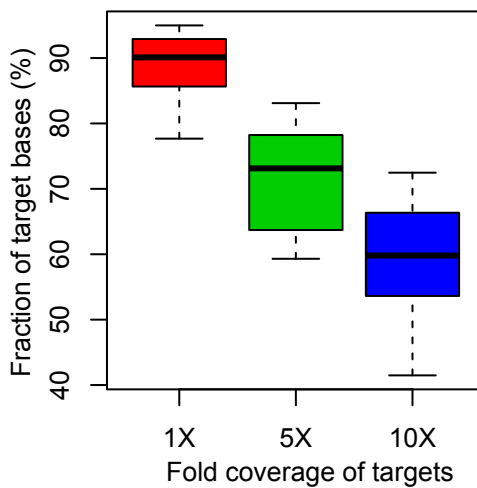

Supplement: Additional file 3 — Figure S2. Fold coverage of target regions for single cells and matched tissues sequenced in the Discovery Screen. (A). The box plot depicted the distribution of mean coverage of tissue from cancer and matched normal tissue sequenced in the discovery stage. CT, cancer tissue; NT, normal tissue. (B). The box plot depicted the distribution of fraction of targeted bases covered by at least 1×, 5× and 10 × across the cancer tissue. (C). The box plot depicted the distribution of mean coverage of all cells from cancer and matched normal sequenced in the discovery stage. Lines in the two central boxes showed the medians, and lines outside the two central boxes showed the first and the third quartiles of the mean depths. SCC, single cells from cancer; SCN, single cells from normal. (D). The box plot depicted the distribution of fraction of targeted bases covered by at least 1×, 5× and 10 × across the 44 qualified cancer cells. Lines in the inner three boxes showed the medians, and lines outside the three boxes showed the first and the third quartiles. [file 2047-217X-1-12-S3.pdf]

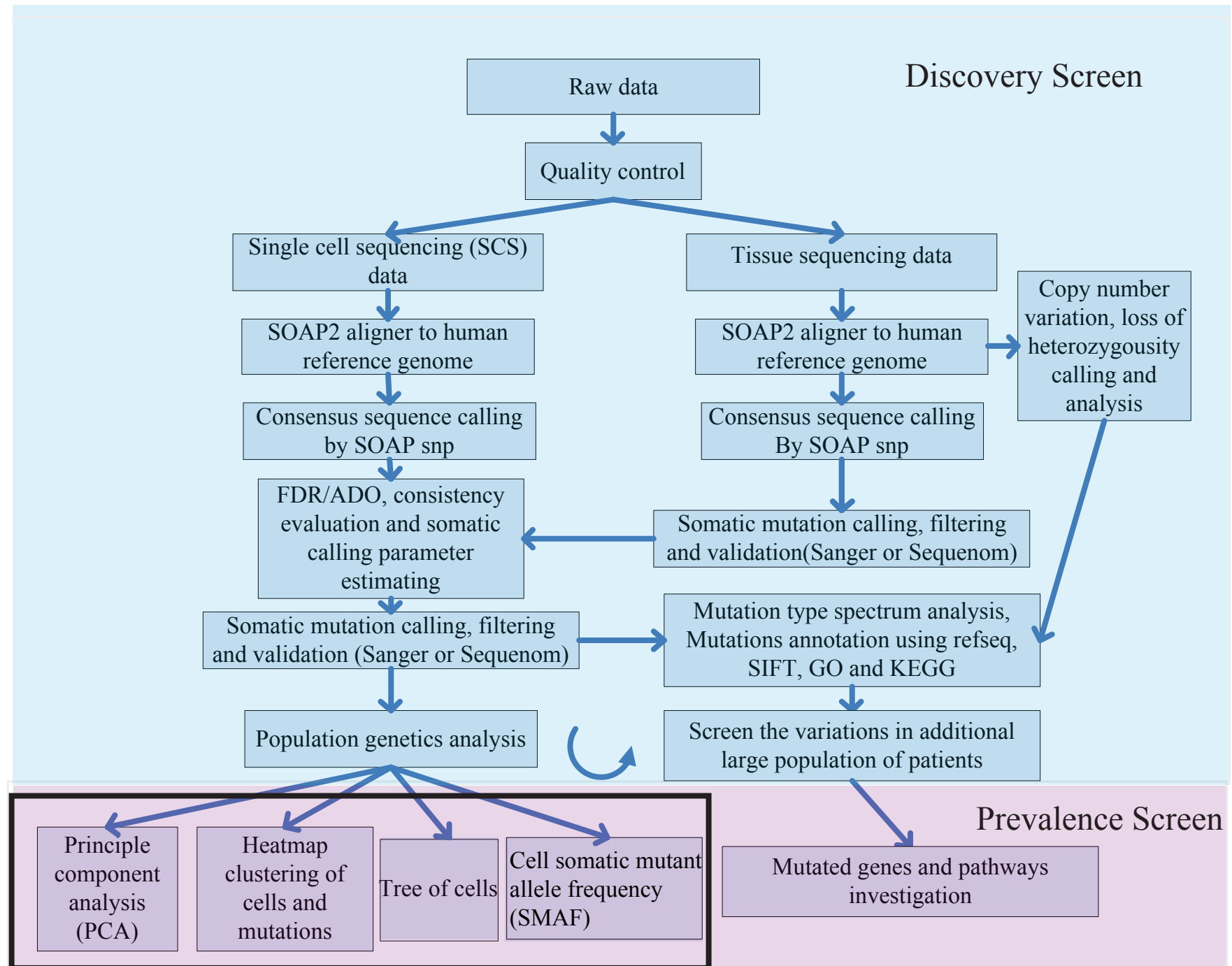

Supplement: Additional file 4 — Figure S3. Bioinformatics pipeline of single cell analyses. [file 2047-217X-1-12-S4.pdf]

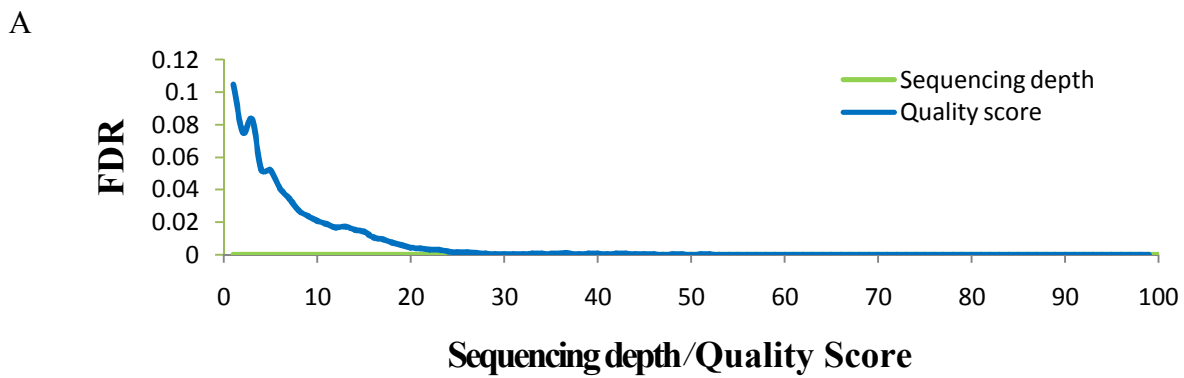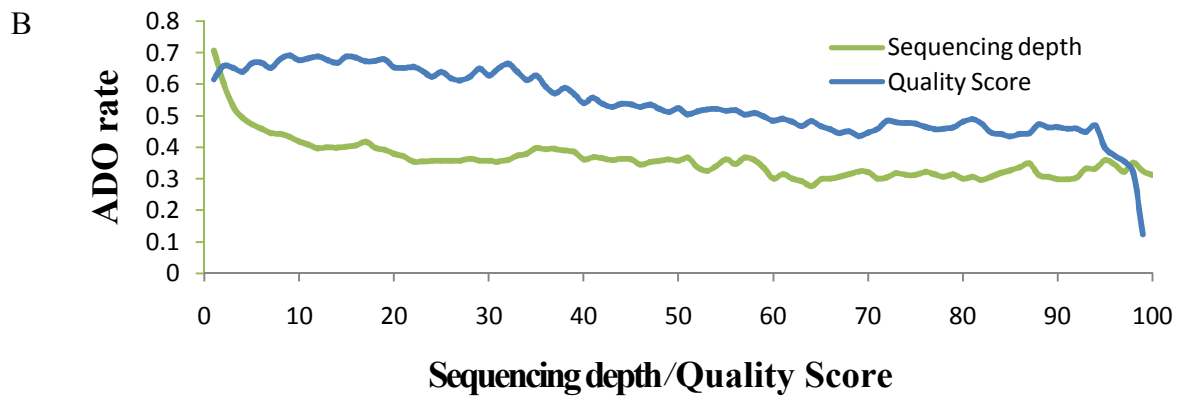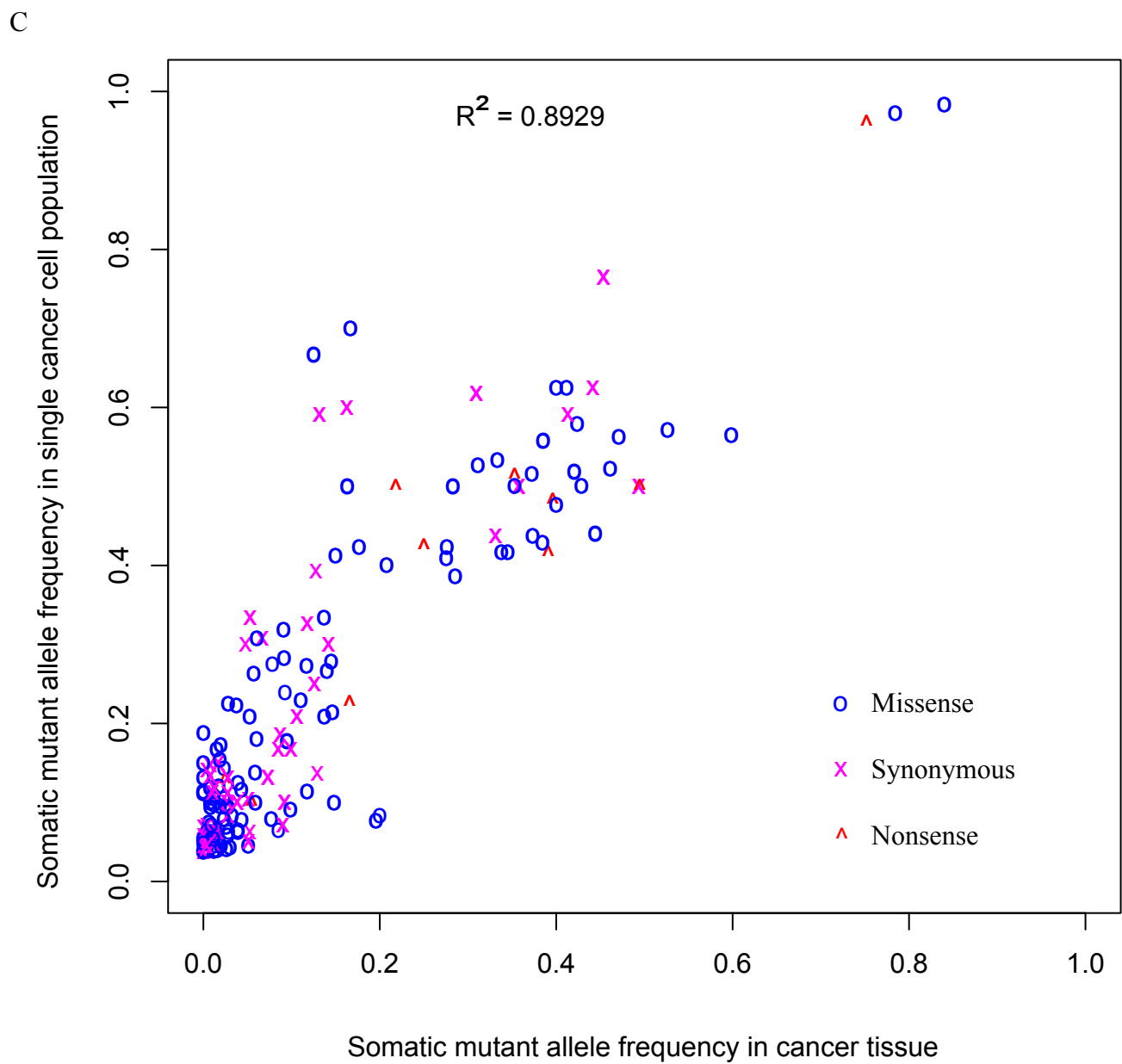

Supplement: Additional file 6 — Figure S4. Quality assessment of single-cell sequencing. (A) and (B). Relationship between estimated false discovery rate (FDR) / allele drop-out (ADO) rates with quality score and read depth after sequencing read alignment in SCS. The selected thresholds (Q20 with depth≥6) in a single cell would result in 0.41 ADO rate and 6.7E-05 FDR. (A). The FDR varied along with the sequencing depth and quality score respectively. (B). The ADO rate varied along with the sequencing depth and quality score respectively. (C). Correlation of frequency between single-cell mutant allele count and mutant allele count in whole tissue sequencing. The single-cell allele count was calculated from the haploids that harbor mutant alleles divided by the total number of haploids (number of cells times 2). The allele count in whole tissue sequencing was calculated by reads harboring mutant alleles divided by total reads covering a site. [file 2047-217X-1-12-S6.pdf]

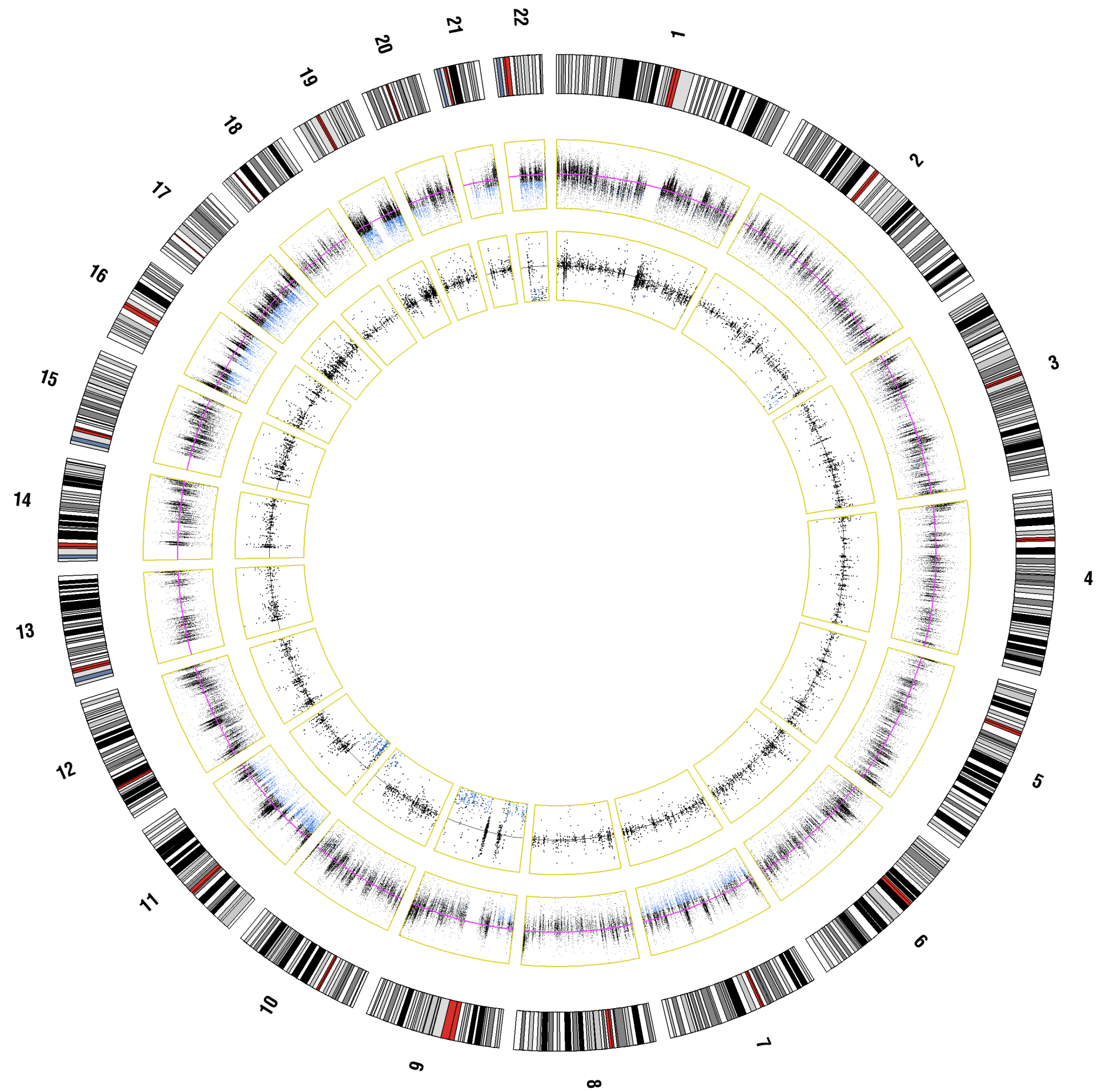

Supplement: Additional file 7 — Figure S5. Copy number variation (CNV) and loss of heterozygosity (LOH) analysis in tissue sequencing of TCC and its matched control across the targeted regions. The CNV and LOH were analyzed with ExomeCNV [15] with default parameters. The most outer ring showed the chromosome ideograms in a pter–qter orientation, clockwise with the centromeres in red. From inside to outside, each data track represented (without Chromosome X and Y): The middle cycle: log ratio of tumor and normal depth-of-coverage, with the segment mean in pink line, the region of gain highlighted in red, and the region of loss highlighted in blue; The inner cycle: the B-allele frequencies (BAF) from ExomeCNV output from tissue exome sequencing with the region of LOH highlighted in blue. [file 2047-217X-1-12-S7.pdf]

# Probabilistic PCA Missing Value Estimator

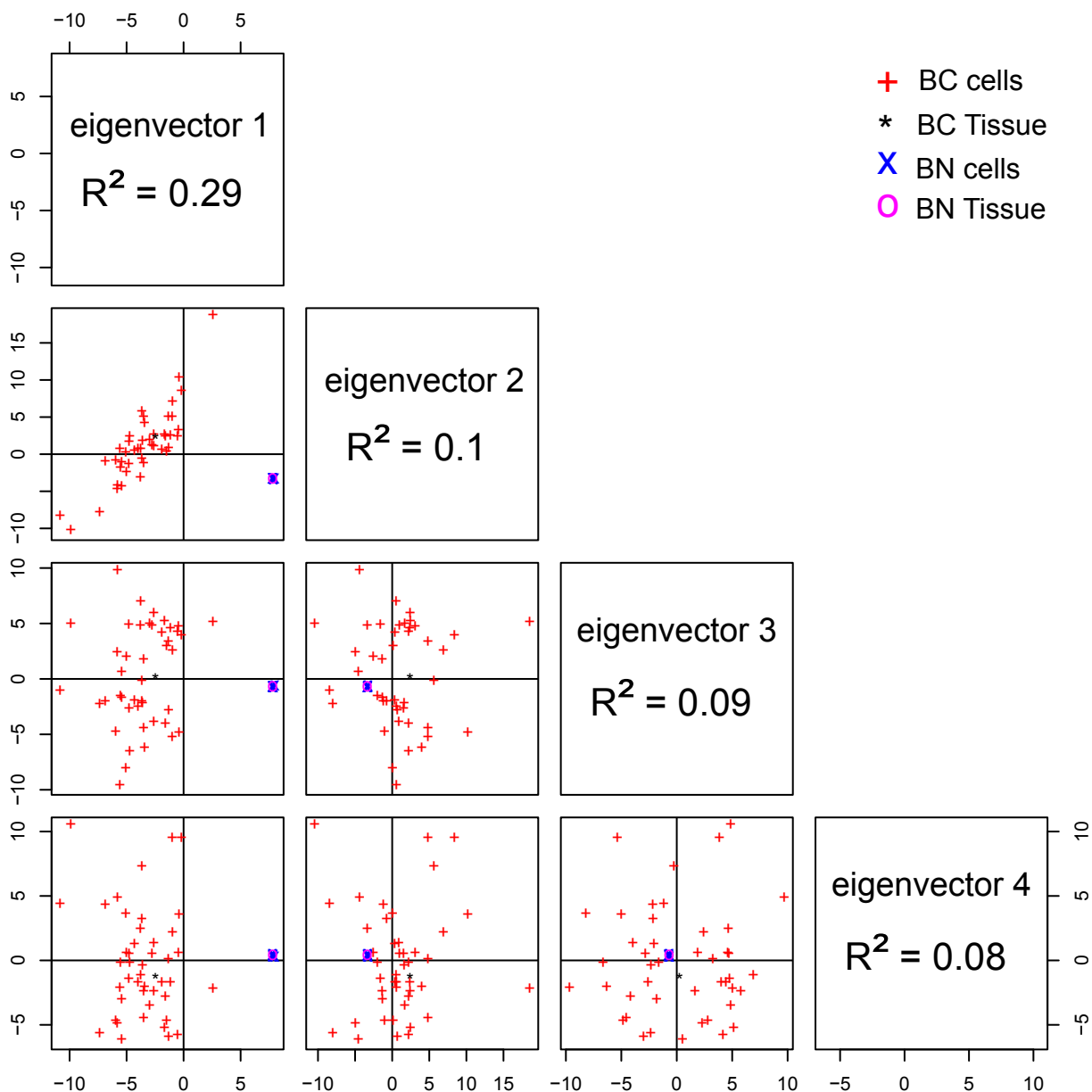

Supplement: Additional file 8 — Figure S6. Principal component analysis (PCA) based on mutant alleles divided tumor cells and normal cells. Since normal cells were genetically extremely close to each other, they were not distinguishable and shown as one point. The exome of BC tissue (purple asterisk) and BN tissue (red tip) were also shown in the PCA to indicate averaged signals. [file 2047-217X-1-12-S8.pdf]

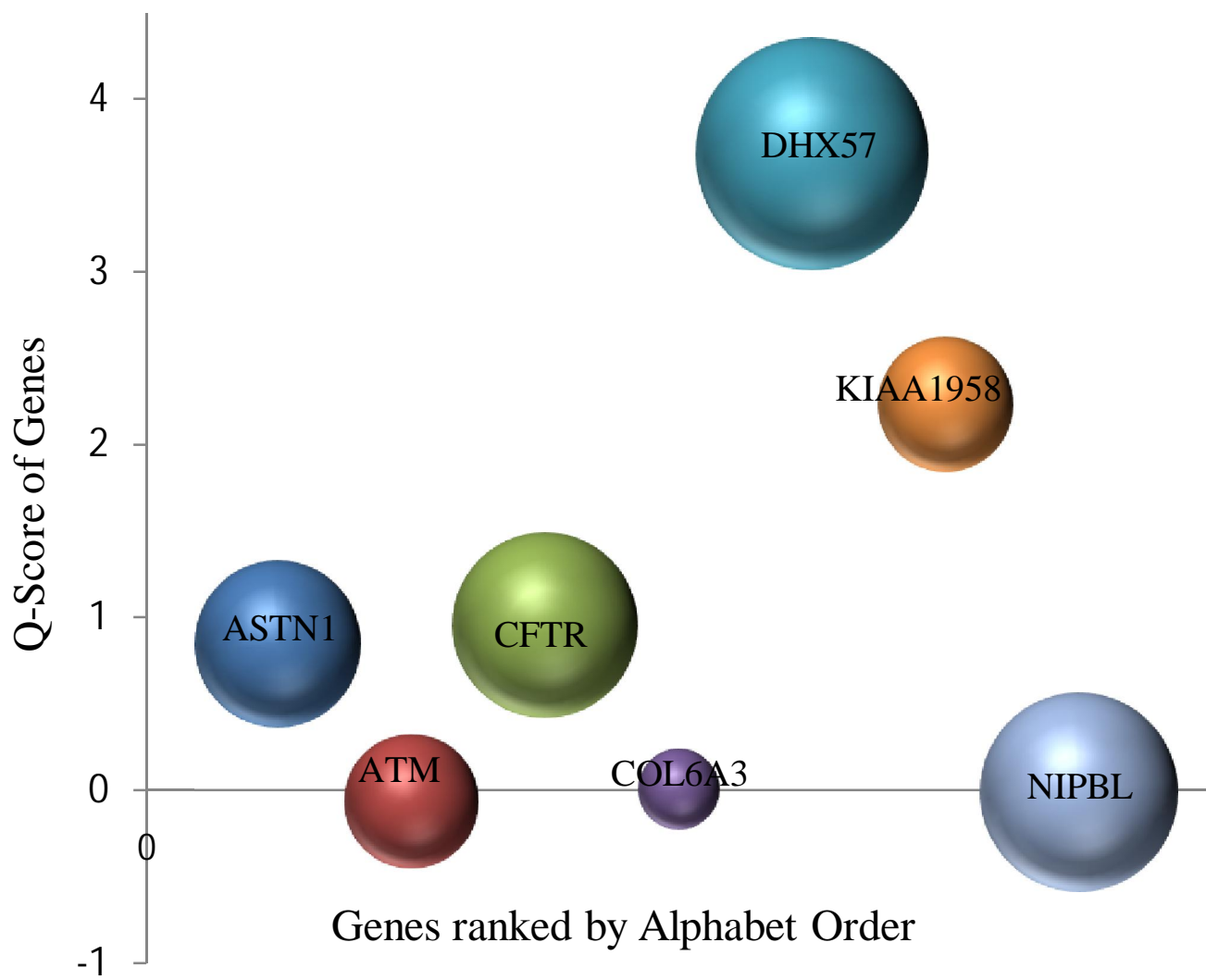

Supplement: Additional file 10 — Figure S7. Driver gene prediction of the recurrent Genes of the TCC. The driver gene prediction analysis of the 7 recurrent genes was indicated as Q-score. The vertical axis was the Q-score, and the circle area indicated the cell mutation frequency. [file 2047-217X-1-12-S10.pdf]
